# Sex Differences in the Association Between Serum Testosterone and Kidney Function in the General Population

**DOI:** 10.1016/j.ekir.2023.04.015

**Published:** 2023-04-24

**Authors:** Anna C. van der Burgh, Elif Aribas, M. Arfan Ikram, Maryam Kavousi, Sebastian J.C.M.M. Neggers, Ewout J. Hoorn, Layal Chaker

**Affiliations:** 1 Department of Internal Medicine, Erasmus University Medical Center, Rotterdam, the Netherlands; 2 Department of Epidemiology, Erasmus University Medical Center, Rotterdam, the Netherlands

**Keywords:** albumin-to-creatinine ratio, estimated glomerular filtration rate, general population, longitudinal, testosterone, sex differences

## Abstract

**Introduction:**

Testosterone might prevent kidney function decline, although evidence is limited in men and lacking in women from the general population. We investigated the association between serum testosterone and kidney function in men and women from a large population-based cohort study.

**Methods:**

Participants aged ≥45 years with available measurements of serum testosterone, sex hormone-binding globulin (SHBG), creatinine, and cystatine C were included. Assessments of kidney function included baseline assessments of the estimated glomerular filtration rate (eGFR) based on serum creatinine (eGFRcreat) or serum cystatin C (eGFRcys), and the urine albumin-to-creatinine ratio (ACR), and repeated assessments of eGFRcreat. Linear regression and linear mixed models were used to assess the associations of serum free and total testosterone with kidney function, stratified for sex.

**Results:**

A total of 4095 men and 5389 women (mean age 65.2 years) were included. In men, higher free testosterone was associated with lower eGFRcreat (beta −0.63, 95% confidence interval [CI]: −1.05; −0.21), higher eGFRcys (beta 0.56, 95% CI: 0.07; 1.05), and lower ACR (beta −0.25, 95% CI: −0.35; −0.16) at baseline. Higher total testosterone was associated with higher baseline and follow-up eGFRcreat, and with lower eGFRcreat when additionally adjusted for SHBG. In women, higher free testosterone was associated with lower baseline eGFRcreat and eGFRcys (beta −1.03, 95% CI: −1.36; −0.71; beta −1.07, 95% CI: −1.44; −0.70; respectively) and lower eGFRcreat over time (beta −0.78, 95% CI: −1.10; −0.46), but not with ACR.

**Conclusions:**

eGFRcys might be a better parameter than eGFRcreat for the association of testosterone with kidney function, although further studies investigating this are needed. Furthermore, we identified sex differences in the association between testosterone and kidney function, with a positive association in men and a negative association in women.

## Graphical abstract

**Figure:**
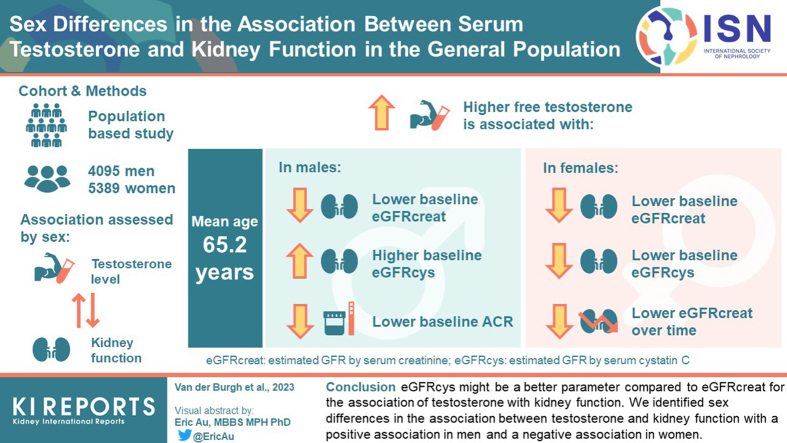

The rate of kidney function decline and the prevalence and prognosis of chronic kidney disease (CKD) is sex-dependent.^1^ Previously, we have shown that the rate of kidney function decline with age differs between men and women from the general population.^2^ The exact underlying mechanisms explaining these differences are unknown; however, sex hormones might play an important role.

Testosterone is the most important sex hormone in men and low testosterone levels have been shown to negatively affect the occurrence of cardiovascular events and mortality in patients with CKD.^3^ It has been implicated that testosterone might have positive effects on the kidney, potentially through inducing vasodilatation in the kidney vascular bed^4^ and reducing kidney inflammation.^5^^,^^6^ However, it is still largely unknown whether testosterone is also associated with positive effects on kidney function in a general population. This knowledge could be instrumental in identifying potential target populations for prevention and treatment strategies for CKD, for example men with hypogonadism. Furthermore, this knowledge could provide more insights into which populations are more protected against kidney function decline and CKD, such as individuals with increased levels of testosterone. An example of these individuals are women with hyperandrogenism, a key feature of polycystic ovary syndrome.^7^

In a recent meta-analysis, only 1 study investigating the association between serum testosterone and kidney function in a general population was identified, which only included men and used eGFRcreat only.^3^ However, eGFRcreat might not be the optimal parameter to analyze the association between testosterone and kidney function because testosterone displays a strong influence on muscle mass and tubular creatinine secretion.^8^, ^9^, ^10^ Because eGFR based on serum cystatin C is less influenced by changes in muscle mass, the use of this parameter might be preferred when analyzing the association between testosterone and kidney function. Therefore, in the current study, we aim to investigate the association between testosterone status and different assessments of kidney function in men and women from the general population in order to provide more insight into the effects of testosterone on kidney function in both sexes and explore whether the associations differ depending on the used assessment of kidney function.

## Methods

### Design and Population

The Rotterdam Study is a prospective population-based cohort study that started in 1990 with the goal of investigating the determinants and occurrence of cardiovascular, neurologic, ophthalmologic, psychiatric, and endocrine diseases in the middle-aged and elderly from the general population. Initially, all inhabitants aged 55 years and older from Ommoord, a suburb in Rotterdam, the Netherlands, were invited to participate, and this initial cohort consisted of 7983 participants. Two additional cohorts were added in 2000, including 3011 participants aged 55 years and older, and in 2006, including 3932 participants aged 45 years and older. By the end of 2008, 14,926 participants aged 45 years and older were included in the study, reflecting an average response rate of 72%. Further details regarding the design and rationale of the Rotterdam Study have been extensively described previously.^11^ Participants from the Rotterdam Study were eligible for the current study if measurements of serum testosterone, serum SHBG, serum creatinine, and serum cystatin C were available at baseline, which was defined as the third visit of the first cohort (1997–1999), the first visit of second cohort (2000–2001), and the first visit of the third cohort (2006–2008). The Rotterdam Study has been approved by the Medical Ethics Committee of the Erasmus MC (registration number MEC 02.1015) and by the Dutch Ministry of Health, Welfare, and Sport (Population Screening Act WBO, license number 1071272-159521-PG). The Rotterdam Study has been entered into the Netherlands National Trial Register (NTR; www.trialregister.nl) and into the WHO International Clinical Trials Registry Platform (ICTRP; www.who.int/ictrp/network/primary/en/) under shared catalog number NTR6831. All included participants provided written informed consent to participate in the study and to have their information obtained from treating physicians.

### Assessment of Testosterone Status

Serum total testosterone was measured using liquid chromatography-tandem mass spectrometry. Serum SHBG was measured using the Immulite platform (Diagnostics Products Corporation Breda, the Netherlands). The interassay coefficients of variations for both serum total testosterone and serum SHBG are <5%. Serum (calculated) free testosterone was determined using the formula described by Vermeulen *et al.*^12^ For calculations, serum albumin was held constant at 4.3 g/dl, because no serum albumin measurements were available.

### Assessment of Kidney Function

Serum creatinine (μmol/l) was measured using an enzymatic assay method. Serum creatinine measurements taken within the Rotterdam Study were supplemented with measurements from the Star-MDC database, which is a database from a center for medical diagnostics for outpatients in the city of Rotterdam.^2^ Serum cystatin C (mg/l) was measured only at baseline using a particle-enhanced immunonephelometric assay. Both eGFRcreat and eGFRcys were calculated according to the Chronic Kidney Disease Epidemiology Collaboration 2012 equation (Supplementary Methods).^13^ Urine albumin and creatinine measurements were available within a subset of the population and were determined in overnight urine samples using a turbidimetric and enzymatic method, respectively, and measured by a Hitachi Modular P analyzer (Roche/Hitachi Diagnostics, Mannheim, Germany). ACR was estimated by dividing urine albumin by urine creatinine (mg/g).

## Assessment of Covariates

At baseline, interviews were performed to obtain information on medical history, medication use, alcohol intake, and smoking. Alcohol intake was recorded in grams per day and smoking was categorized into never, past, and current smoking. Use of lipid-lowering medication and use of sex hormones and modulators of the genital system were defined using the WHO’s Anatomical Therapeutic Chemical codes C10 and G03, respectively. Weight and height were measured at the research center and body mass index was calculated by dividing weight in kilograms by height in meters squared (kg/m^2^). Systolic and diastolic blood pressure were measured in a sitting position using a random-zero sphygmomanometer and the mean of 2 consecutive measurements was taken as the final measurement. Hypertension was defined as a systolic blood pressure ≥140 mm Hg, a diastolic blood pressure of ≥90 mm Hg, or the use of antihypertensive drugs. Serum cholesterol (mmol/l), thyroid-stimulating hormone (mU/l) and C-reactive protein (mg/ml) levels were measured by the Department of Clinical Chemistry of the Erasmus Medical Center using standard laboratory techniques. Diabetes mellitus was defined as a fasting serum glucose level of at least 7 mmol/l, a nonfasting serum glucose level of at least 11.1 mmol/l (if fasting glucose was not present), the use of antidiabetic medication, or a previous diagnosis of the disease. History of cardiovascular disease (CVD), defined as a history of myocardial infarction, stroke, and coronary or other arterial revascularization, was assessed during the baseline home interviews, and verified using clinical data from the medical records. Appendicular lean mass, defined as the sum of the lean tissue from the arms and legs, was measured using dual-energy X-ray absorptiometry with an iDXA total body fan-beam densitometer (GE Lunar Corp., Madison, WI, USA). The skeletal muscle index (SMI), adjusted for variation in skeletal size, was defined as appendicular lean mass divided by height in meters squared (kg/m^2^) and was used as a measure of muscle mass.

### Statistical Analysis

All analyses were performed for men and women separately. Linear regression analyses were conducted to study the associations of baseline serum free and total testosterone with eGFRcys and eGFRcreat. Linear regression analyses were also conducted to study the association of SHBG with eGFRcys and eGFRcreat, as well as with serum creatinine and cystatin C. Linear regression assumptions were met for all analyses. Results were reported as standardized betas (beta per SD of the determinant) with their 95% CI to compare the effect of the determinants on the outcome. The associations of serum total and free testosterone with repeated assessments of eGFRcreat were studied using linear mixed models^14^ in which time of kidney function assessment was used as the time variable. A nonlinear effect of time was taken into account using natural cubic splines with 3 knots because this provided the best fit with the data. Random effects (i.e., for individual participants) of the linear mixed models included random intercepts and nonlinear random slopes (i.e., of time). Linear regression analyses were used to study the association of serum free and total testosterone with urine ACR. Urine ACR was not normally distributed and therefore, a natural log-transformation was used. We added 1 mg/g to the nontransformed values to account for zero values of urine ACR. For the analyses using serum free testosterone as a determinant, we used 3 models. In the first model, we adjusted for age and Rotterdam Study Cohort. Age is included in the formula for calculating eGFR, however this does not automatically take age into account as a potential confounder of the exposure-outcome association. Therefore, we adjusted for age in our first model. In the second model, we additionally adjusted the first model for the potential confounders, including smoking, alcohol use, and thyroid-stimulating hormone. In the third model, we additionally adjusted the second model for potential confounders which could also acts as mediators, including serum cholesterol, serum C-reactive protein, body mass index, hypertension, and diabetes mellitus. To explore the role of muscle mass within the association between testosterone and kidney function, we additionally adjusted the second model for SMI and investigated potential effect modification by adding an interaction term between testosterone and SMI to the models. For the analyses regarding serum total testosterone, a fourth model was added in which we additionally adjusted the second model for SHBG. In sensitivity analyses, we excluded participants using lipid-lowering medication and participants using sex hormones and modulators of the genital system. In addition, we restricted our analyses to participants with serum free and total testosterone within the reference range. The reference range was calculated using the 2.5^th^ and 97.5^th^ percentile and compared to the reference range noted for the used assays. Predefined stratification by age at baseline was performed and reported for serum free testosterone only, because this is the biologically active component of the hormone. We defined the cut-off values for age as 55 and 65 years, because these are the ages often used to define the start of late menopause and the median of our population, respectively.

Missing values in covariates (missingness for all variables <3%, except for alcohol use [20%] and serum C-reactive protein [38%]) were handled by multiple imputation using the Multivariate Imputation by Chained Equations package in R. Statistical analyses were performed using R statistical software (R-project, R Foundation for Statistical Computing [2020], 3.6.3).

## Results

We included 9484 participants (Supplementary Figure S1) with a mean age of 65.2 years, of whom 56.8% were women. Men had a higher prevalence of hypertension, diabetes, and CVD than women (Table 1). In addition, men were more often past smokers and used a higher amount of alcohol per day. The *P* for interaction between testosterone and sex was <0.001, for both free and total testosterone and with all included kidney outcomes. Assessments of eGFRcreat over time were collected during a median follow-up time of 7.8 years (interquartile range 5.5; 13.0) for males and of 9.4 years (interquartile range 5.7; 14.1) for females.

**Table 1:** Baseline characteristics, separately for men and women

Variable	Men (*n* = 4095)	Women (*n* = 5389)	Missing data (*n* men/*n* women)
Age, yr (*n* = 4095; *n* = 5389)	64.7 ± 9.4	65.6 ± 10.2	0/0
Body mass index, kg/m^2^ (*n* = 4060; *n* = 5288)	27.0 ± 3.6	27.4 ± 4.6	35/101
Systolic blood pressure, mm Hg (*n* = 4082; *n* = 5348)	141 ± 20	139 ± 22	13/41
Diastolic blood pressure, mm Hg (*n* = 4082; *n* = 5348)	80 ± 12	78 ± 11	13/41
Hypertension, *n* (valid%) (*n* = 4029; *n* = 5264)	2106 (52.3)	2529 (48.0)	66/125
Diabetes mellitus, *n* (%) (*n* = 4095; *n* = 5389)	599 (14.6)	559 (10.4)	0/0
History of CVD, *n* (valid%) (*n* = 4068; *n* = 5365)	628 (15.4)	303 (5.6)	27/24
Smoking (*n* = 4069; *n* = 5316)			26/73
Current smoking, *n* (valid%)	813 (20.0)	1005 (18.9)	
Past smoking, *n* (valid%)	2451 (60.2)	2050 (38.6)	
Never smoking, *n* (valid%)	805 (19.8)	2261 (42.5)	
Alcohol use, g/d (*n* = 3274; *n* = 4461)	8.6 (1.6–20.0)	1.6 (0.3–8.6)	
eGFR creatinine, ml/min per 1.73 m^2^ (*n* = 4095; *n* = 5389)	80 ± 15	79 ± 15	0/0
eGFR cystatin C, ml/min per 1.73 m^2^ (*n* = 4095; *n* = 5389)	78 ± 19	76 ± 19	0/0
Serum cholesterol, mmol/l (*n* = 4085; *n* = 5341)	5.5 ± 1.0	5.9 ± 1.0	10/48
Serum TSH, mU/l (*n* = 4095; *n* = 5387)	2.18 ± 2.85	2.60 ± 3.49	0/2
Serum CRP, mg/l (*n* = 2533; *n* = 3190)	2.4 ± 4.7	2.6 ± 4.4	1562/2199
Serum SHBG, nmol/l (*n* = 4095; *n* = 5389)	47.7 ± 20.2	67.2 ± 34.1	0/0
Serum testosterone, nmol/l (*n* = 4095; *n* = 5389)	17.2 ± 6.0	0.9 ± 0.8	0/0
Serum free testosterone^a^, nmol/l (*n* = 4095; *n* = 5389)	0.29 ± 0.09	0.01 ± 0.01	0/0
Skeletal muscle index (*n* = 2436; *n* = 3346)	8.35 ± 0.85	6.62 ± 0.70	1659/2043

CRP, C-reactive protein; CVD, cardiovascular disease; eGFR, estimated glomular filtration rate; *n*, number; SHBG, sex-hormone binding globulin; TSH, thyroid-stimulating hormone.

Data are presented as number (%), number (valid%), mean ± SD, or median (interquartile range). Values are shown for nonimputed data. For variables with missing data, valid % is given. Number of complete observations is represented by n, for men and women, respectively.

a Calculated using serum total testosterone, serum SHBG, and constant serum albumin of 4.3 g/dl.

### Serum Free and Total Testosterone and Kidney Function in Men

#### Free Testosterone and eGFR

The total study population included 4095 men. In men, higher levels of serum free testosterone were associated with lower levels of eGFRcreat at baseline (beta −0.63, 95% CI: −1.05; −0.21, model 2); however not with eGFRcreat over time (beta −0.36, 95% CI: −0.75; 0.04, model 2) (Table 2). The association between serum free testosterone and eGFRcreat over time did reach statistical significance after adjustment for serum cholesterol, serum C-reactive protein, body mass index, hypertension, and diabetes (beta −0.57, 95% CI: −0.97; −0.17, model 3, Supplementary Table S1). In contrast, higher levels of serum free testosterone were significantly associated with higher levels of eGFRcys at baseline (beta 0.56, 95% CI: 0.07; 1.05, model 2, Table 2). When excluding participants with serum free testosterone outside the reference range (*n* = 3889), the association of serum free testosterone with eGFRcreat at baseline lost significance (beta −0.48, 95% CI: −1.00; 0.03, Supplementary Table S2), whereas the association of free testosterone with eGFRcreat and eGFRcys did not change substantially. Other additional adjustments and sensitivity analyses did not substantially change the results (Supplementary Tables S1 and S3). No significant interaction between eGFR and SMI was identified (*P* for interaction > 0.10 for both eGFRcreat and eGFRcys).

**Table 2:** Association of standardized serum free testosterone with eGFRcreat, eGFRcys, and the log-transformed ACR (mg/g), separately for men and women

Subgroup	Outcome	Beta (95% CI), model 1	Beta (95% CI), model 2
Men (*n* = 4095)	eGFRcreat, baseline	−0.61 (−1.03; −0.19)	−0.63 (−1.05; −0.21)
	eGFRcreat, repeated	−0.34 (−0.74; −0.05)	−0.36 (−0.75; 0.04)
	eGFRcys, baseline	0.54 (0.05; 1.04)	0.56 (0.07; 1.05)
	ACR, baseline^a^	−0.27 (−0.38; −0.16)	−0.25 (−0.35; −0.16)
Women (*n* = 5389)	eGFRcreat, baseline	−1.03 (−1.36; −0.70)	−1.03 (−1.36; −0.71)
	eGFRcreat, repeated	−0.77 (−1.09; −0.45)	−0.78 (−1.10; −0.46)
	eGFRcys, baseline	−1.05 (−1.42; −0.69)	−1.07 (−1.44; −0.70)
	ACR, baseline^a^	−0.21 (−0.99; 0.56)	−0.26 (−1.03; 0.51)

ACR, albumin-to-creatinine ratio; CI, confidence interval; eGFRcreat, estimated glomerular filtration rate based on serum creatinine; eGFRcys, estimated glomerular filtration based on serum cystatin C; n, number.

Model 1 is adjusted for age at baseline and Rotterdam Study Cohort. Model 2 is additionally adjusted for smoking, alcohol use, and TSH.

a Available in a subset of the population; *n* = 1359 for men and *n* = 1773 for women.

#### Total Testosterone and eGFR

Higher levels of serum total testosterone were significantly associated with higher levels of eGFRcreat at baseline (beta 0.48, 95% CI: 0.09; 0.87, model 2), as well as with higher levels of eGFRcreat over time (beta 0.76, 95% CI: 0.39; 1.13, model 2) (Supplementary Table S1). When additionally adjusting for serum SHBG, the directionality of the association changed and higher levels of serum total testosterone were associated with lower levels of eGFRcreat at baseline (beta −0.73, 95% CI: −1.23; −0.22, model 4) and over time (beta −0.42, 95% CI: −0.89; 0.05) (Supplementary Table S1). In contrast, higher levels of serum total testosterone were associated with higher levels of eGFRcys at baseline when adjusting for potential confounders and serum SHBG (beta 0.60, 95% CI: 0.01; 1.19, model 4, Supplementary Table S1).

#### SHBG and eGFR

Higher levels of serum SHBG were significantly associated with higher levels of eGFRcreat (beta 1.49, 95% CI: 1.08; 1.90, model 2, Supplementary Table S4), whereas no significant association with eGFRcys was reported. Higher levels of serum SHBG were significantly associated with lower levels of serum creatinine (beta −0.03, 95% CI: −0.04; −0.02, model 2, Supplementary Table S4), but not with serum cystatin C.

#### Free and Total Testosterone and ACR

Data on the urine ACR was available for 1359 men, with a median ACR of 2.90 (interquartile range 1.85; 5.68). Higher levels of serum free testosterone were associated with significantly lower ACR levels when correcting for potential confounders (beta −0.25, 95% CI: −0.35; −0.16, model 2, Table 2). Similar results were reported with total testosterone (Supplementary Table S5).

### Serum Free and Total Testosterone and Kidney Function in Women

#### Free and Total Testosterone and eGFR

A total number of 5389 women were included in the study. In women, higher levels of serum free testosterone were significantly associated with lower levels of eGFRcreat and eGFRcys at baseline (beta −1.03, 95% CI: −1.36; −0.71 and beta −1.07, 95% CI: −1.44; −0.70, respectively, model 2), as well as with lower levels of eGFRcreat over time (beta −0.78, 95% CI: −1.10; −0.46, model 2) (Table 2). Additional adjustments and sensitivity analyses did not substantially change results (Supplementary Tables S1–S3). No significant interaction between eGFR and SMI was identified (*P* for interaction > 0.10 for both eGFRcreat and eGFRcys). Similar results were found for serum total testosterone (Supplementary Table S1).

#### Free and Total Testosterone and ACR

Data on the urine ACR was available for 1773 women, with a median ACR of 3.90 (interquartile range 2.53; 6.81). Serum free testosterone was not significantly associated with ACR (beta −0.26, 95% CI: −1.03; 0.51, Table 2). Similar results were reported with serum total testosterone (Supplementary Table S5).

### Stratified Analyses by Age in Men and Women

**Figure 1:**
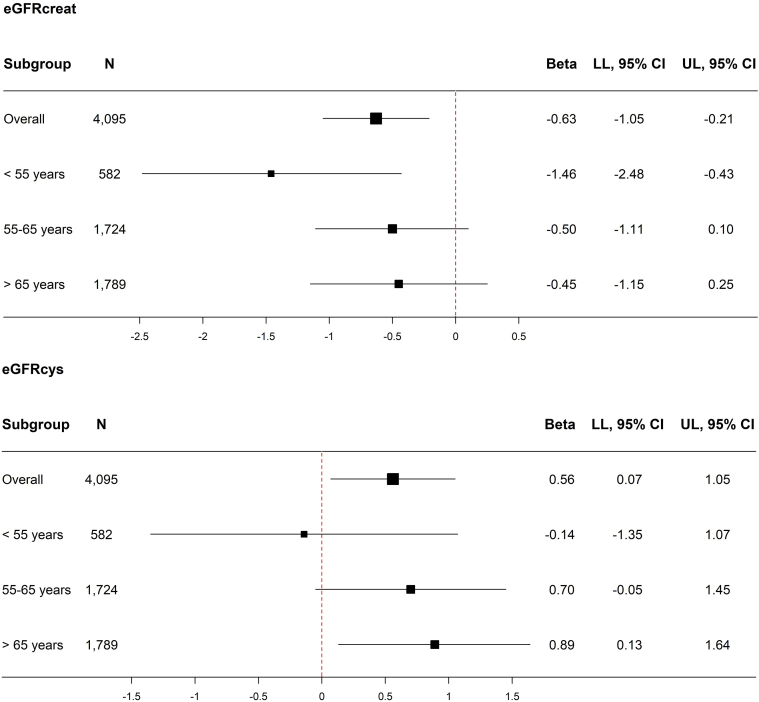
Stratified analyses for the association between serum free testosterone and eGFRcreat and eGFRcys at baseline in men, overall and stratified on baseline age. Analyses are adjusted for age at baseline, Rotterdam Study Cohort, smoking, alcohol use, and TSH. The *P* for interaction of serum free testosterone and age is 0.06 with eGFRcreat as the outcome and <0.01 with eGFRcys as the outcome. CI, confidence interval; eGFRcreat, estimated glomerular filtration rate based on serum creatinine; eGFRcys, estimated glomerular filtration based on serum cystatin C; LL, lower limit; n, number; TSH, thyroid-stimulating hormone; UL, upper limit.

**Figure 2:**
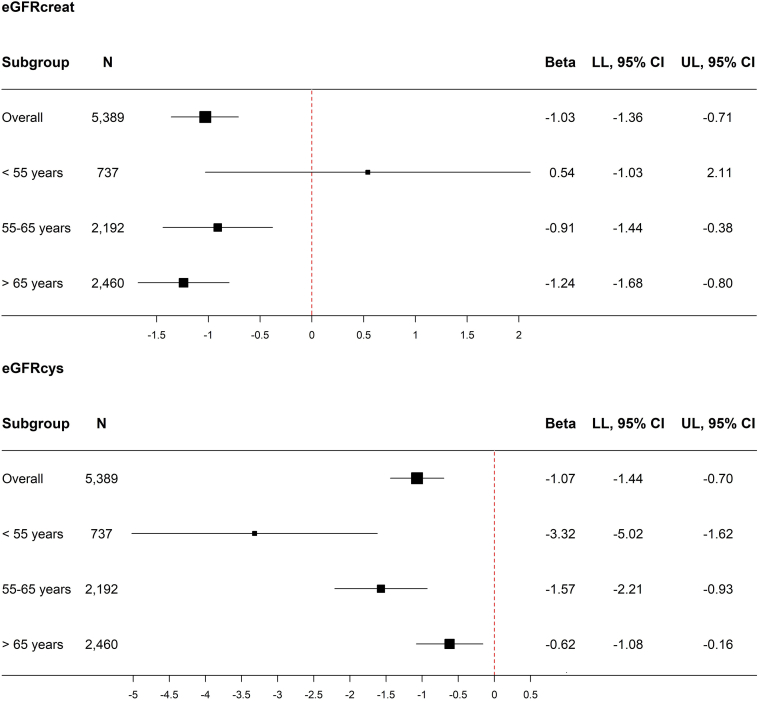
Stratified analyses for the association between serum free testosterone and eGFRcreat and eGFRcys at baseline in women, overall and stratified on baseline age. Analyses are adjusted for age at baseline, Rotterdam Study Cohort, smoking, alcohol use, and TSH. The *P* for interaction of serum free testosterone and age is 0.03 with eGFRcreat as the outcome and <0.01 with eGFRcys as the outcome. CI, confidence interval; eGFRcreat, estimated glomerular filtration rate based on serum creatinine; eGFRcys, estimated glomerular filtration based on serum cystatin C; LL, lower limit; n, number; TSH, thyroid-stimulating hormone; UL, upper limit.

When stratifying by age in men, higher levels of serum free testosterone were only significantly associated with lower levels of eGFRcreat in participants who were younger than 55 years and with higher levels of eGFRcys in participants who were aged above 65 years (Figure 1). However, the beta of the association was similar to the beta reported in participants aged between 55 and 65 years. When stratifying by age in women, higher levels of serum free testosterone were significantly associated with lower levels of eGFRcreat in all age categories except for the age category including participants who were younger than 55 years (Figure 2). In contrast, higher levels of serum free testosterone were significantly associated with lower levels of eGFRcys in all age categories.

## Discussion

In this population-based cohort study, we reported a sex-dependent association between serum testosterone and kidney function. In men, higher free testosterone levels were associated with higher eGFRcys and lower ACR. Conversely, higher free testosterone levels were associated with lower eGFRcreat. Higher total testosterone levels, unadjusted for SHBG, were associated with higher eGFRcreat and lower ACR. In women, higher levels of free and total testosterone were associated with lower eGFRcreat and eGFRcys, whereas no association with ACR was shown. The identified sex differences may suggest that testosterone is detrimental to kidney function in women but beneficial for kidney function in men.

Detrimental effects, however, were only reported in animal studies, which investigated testosterone depletion with or without consecutive testosterone replacement, and included glomerular and tubular damage, kidney fibrosis, proteinuria, and hypertensive effects.^15^, ^16^, ^17^ Interestingly, beneficial effects were most often reported in men, whereas detrimental effects were reported in both sexes and in animals. In women, higher serum testosterone has been associated with a negative cardiovascular risk profile^18^, ^19^, ^20^ and increased risk of CVD,^21^^,^^22^ even though an increased risk of CVD with low serum testosterone levels has been suggested as well.^21^^,^^23^ In contrast, higher serum testosterone in men has been associated with a better cardiovascular risk profile and lower risk of CVD.^24^, ^25^, ^26^ A previous study investigating the association between testosterone and type 2 diabetes reported similar sex-dependent results.^27^ Of note, however, even after correcting for several cardiovascular risk factors and CVD in our analyses, the sex differences remained, suggesting the presence of other mechanisms or a direct effect of sex hormones on the kidney.

Even though higher levels of testosterone seemed to be beneficial for the kidney in men, different results were reported dependent on the type of assessment of kidney function and testosterone. The reported discrepancy between eGFRcreat on the one hand and eGFRcys and ACR on the other hand suggests that the negative association between serum free testosterone and eGFRcreat is explained by an effect on serum creatinine rather than by an effect on kidney function. A possible explanation is the direct effect of SHGB on serum creatinine. SHBG appears to have functions beyond androgen regulation.^28^ For example, SHBG receptors have previously been identified in sex-steroid dependent tissues such as the endometrium and the prostate, and specific binding to these receptors might have effects on cell growth and biochemical endpoints.^28^, ^29^, ^30^ Whether these receptors are also present on skeletal muscle is still unclear^31^^,^^32^; however, SHBG binding to and action in muscle might explain the association between SHBG and serum creatinine and the discrepancy between eGFRcreat and eGFRcys. Another explanation for this discrepancy might be a mediating role of muscle mass within the association between serum testosterone and eGFRcreat, even though similar results were reported after adjusting for the SMI and no significant interaction between eGFRcreat and SMI was reported. Because serum cystatin C is likely not affected by muscle mass,^33^ it might be superior to serum creatinine as a marker of kidney function when interpreting the association between serum testosterone and kidney function. Another measure of kidney function is the urine ACR, which is only partly affected by differences in muscle mass,^34^ and these results are indeed in line with eGFRcys.

To account for menopause and potentially andropause, we stratified our analyses by age. In men, our results from this age-stratified analysis regarding the association between testosterone and eGFRcreat are of particular interest. We report that higher levels of free testosterone were most relevantly associated with lower levels of eGFRcreat in participants who were younger than 55 years. The explanation for this finding is unknown, although it might be related to the determinants of muscle mass. Before the age of 55 years, testosterone might be the most important determinant of muscle mass and loss, whereas other determinants could play a role at older age as well, including age itself, low body weight, prolonged immobilizations, and reduced protein intake,^35^ The presence of other determinants than testosterone might limit the importance of testosterone as a determinant of muscle mass and loss in older age. In women, higher levels of free testosterone were associated with lower levels of eGFRcreat in all age categories except for the age category including participants younger than 55 years. This could be explained by the menopausal state of the included women. Given that estrogen is suggested to have beneficial effects on the kidney,^1^^,^^36^ the drop in estrogen levels with menopause could take away these positive effects which potentially balance out the negative effects of testosterone in premenopausal women. However, higher levels of free testosterone were significantly associated with lower levels of eGFRcys in participants aged below 55 years, whereas the associations between free testosterone and eGFRcys in participants aged above 55 years were comparable to the associations found between free testosterone and eGFRcreat in the same age groups. In an animal study, estrogen was shown to enhance cystatin C expression in the vagina.^37^ Whether or not estrogen levels affect cystatin C expression and blood level in humans is unknown, although it could potentially explain the differences in results between eGFRcreat and eGFRcys in women aged below 55 years.

Strengths of our study include the large number of participants from a population-based cohort, the availability of several (repeated) measurements of kidney function and serum testosterone, and the ability to adjust for a wide variety of confounders. A limitation of our study is that the included population includes mainly Caucasian individuals aged above 45 years, which might limit the generalizability of our findings to other populations. In addition, serum free testosterone levels were determined assuming a constant serum albumin level, because no serum albumin levels were available in our population. However, serum albumin levels are expected to be relatively stable within the general population. Furthermore, residual confounding cannot be fully excluded despite the large number of variables included in the analyses. Finally, our study included only a small number of participants aged below 55 years, which limited the statistical power to investigate the associations of interest in this group of participants. Therefore, non-significance of our findings in this age group should be interpreted with caution.

In conclusion, our findings suggest that eGFRcreat is not the optimal parameter to investigate the association between testosterone and kidney function. Our findings also suggest that the association between serum testosterone and kidney function is sex-dependent. Our results need to be viewed as a first signal that testosterone may be an interesting biomarker and/or therapeutic target in the context of kidney disease prevention and treatment. Nevertheless, if our findings are replicated and found to be causal, there could be several implications. Higher serum testosterone levels were associated with better kidney function in men. Testosterone might therefore be a modifiable target to prevent or treat low kidney function in men with hypogonadism, although intervention studies are needed to investigate whether treatment with testosterone replacement therapy is beneficial for kidney function in these men. The potential adverse effects of this therapy in especially individuals with already low kidney function should be investigated as well, because fluid retention has been suggested as one of the potential adverse effects.^38^^,^^39^ On the other hand, higher serum testosterone levels were associated with worse kidney function in women. This might indicate that women with hyperandrogenism such as those with polycystic ovary syndrome are at higher risk of having or developing kidney dysfunction and therefore, kidney function monitoring to prevent CKD development might be pivotal in this group of women. However, more extensive research exploring the association between hyperandrogenism and kidney function in these women is first needed.

## Disclosure

The authors have declared no competing interests.

## Supplementary Materials

**Supplementary Methods.**

**Table S1.** Association of standardized serum total and free testosterone with eGFRcreat and eGFRcys, separately for men and women.

**Table S2.** Association of standardized serum free testosterone within the reference range with eGFRcreat and eGFRcys in men and women.

**Table S3.** Association of standardized serum free testosterone with eGFRcreat and eGFRcys in men and women not using lipid-lowering medication or sex hormones and modulators of the genital system.

**Table S4.** Association of standardized serum SHBG with eGFRcreat and eGFRcys, in men.

**Table S5.** Association between standardized serum total testosterone and urine ACR at baseline, separately for men and women.

**Figure S1.** Flowchart of the study selection.

**STROBE** **Statement.**
