# Supplementary material for: Sex Differences in the Association Between Serum Testosterone and Kidney Function in the General Population
Source: Kidney Int Rep. 2023 Apr 24;8(7):1342–51. doi: 10.1016/j.ekir.2023.04.015 (PMC10334405; doi:10.1016/j.ekir.2023.04.015)
Supplement: Supplementary File (PDF) [file mmc1.docx]

**Supplementary Methods.** Equations for estimating GFR based on serum creatinine and cystatin C

CKD-EPI creatinine equation:

141×min(serum creatinine/κ, 1)^α^×max(serum creatinine/κ, 1)^−1.209^×0.993^Age^ [×1.018 if female] [×1.159 if black]

Where:

Min = minimum of serum creatinine/κ or 1

Max = maximum of serum creatinine /κ or 1

Females: κ = 0.7, α = −0.329

Males: κ = 0.9, α = −0.411

CKD-EPI cystatin C equation:

133×min(serum cystatin C/0.8, 1)^−0.499^×max (serum cystatin C /0.8, 1)^−1.328^×0.996^Age^ [×0.932 if female]

Where:

Min = minimum of serum cystatin C /0.8 or 1

Max = maximum of serum cystatin C /0.8 or 1

Reference

*Inker LA, Schmid CH, Tighiouart H, Eckfeldt JH, Feldman HI, Greene T, et al. Estimating glomerular filtration rate from serum creatinine and cystatin C. N Engl J Med. 2012;367(1):20-9.*

**Supplementary Table S1.** Association of standardized serum total and free testosterone with eGFRcreat and eGFRcys, separately for men and women

| **Subgroup** | **Exposure** | **Outcome** | **Beta**  **(95% CI),**  **Model 1** | **Beta**  **(95% CI),**  **Model 2** | **Beta**  **(95% CI),**  **Model 3** | **Beta**  **(95% CI),**  **Model 4** | **Beta**  **(95% CI),**  **Model 5** |
| --- | --- | --- | --- | --- | --- | --- | --- |
| Men  (n = 4095) | Free serum testosterone | eGFRcreat, baseline | -0.61  (-1.03;-0.19) | -0.63  (-1.05;-0.21) | -0.75  (-1.19;-0.32) | *NA* | -0.59  (-1.01;-0.17) |
|  |  | eGFRcreat, repeated | -0.34  (-0.74;0.05) | -0.36  (-0.75;0.04) | -0.57  (-0.97;-0.17) | *NA* | -0.32  (-0.72;0.07) |
|  |  | eGFRcys, baseline | 0.54  (0.05;1.04) | 0.56  (0.07;1.05) | 0.13  (-0.36;0.63) | *NA* | 0.57 (0.08;1.06) |
|  | Total serum testosterone | eGFRcreat, baseline | 0.54  (0.15;0.94) | 0.48  (0.09;0.87) | 0.36  (-0.06;0.77) | -0.73  (-1.23;-0.22) | 0.34  (-0.00;0.77) |
|  |  | eGFRcreat, repeated | 0.78  (0.42;1.15) | 0.76  (0.39;1.13) | 0.53  (0.14;0.91) | -0.42  (-0.89;0.05) | 0.66  (0.30;1.03) |
|  |  | eGFRcys, baseline | -0.03  (-0.49;0.42) | 0.16  (-0.29;0.62) | -0.48  (-0.96;  -0.008) | 0.60  (0.01;1.19) | 0.15  (-0.31;0.61) |
| Women  (n = 5389) | Free serum testosterone | eGFRcreat, baseline | -1.03  (-1.36;-0.70) | -1.03  (-1.36;-0.71) | -1.04  (-1.38;-0.71) | *NA* | -0.89  (-1.21;-0.56) |
|  |  | eGFRcreat, repeated | -0.77  (-1.09;-0.45) | -0.78  (-1.10;-0.46) | -0.68  (-1.01;-0.36) | *NA* | -0.62  (-0.94;-0.30) |
|  |  | eGFRcys, baseline | -1.05  (-1.42;-0.69) | -1.07  (-1.44;-0.70) | -0.68  (-1.04;-0.32) | *NA* | -0.92  (-1.29;-0.55) |
|  | Total serum testosterone | eGFRcreat, baseline | -0.72  (-1.11;-0.45) | -0.77  (-1.10;-0.44) | -0.77  (-1.10;-0.44) | -0.83  (-1.16;-0.49) | -0.78  (-1.11;-0.45) |
|  |  | eGFRcreat, repeated | -0.44  (-0.79;-0.10) | -0.50  (-0.84;-0.16) | -0.49  (-0.83;-0.15) | -0.58  (-0.92;-0.24) | -0.50  (-0.84;-0.17) |
|  |  | eGFRcys, baseline | -0.85  (-1.22;-0.48) | -0.81  (-1.18;-0.44) | -0.82  (-1.18;-0.46) | -0.89  (-1.26;-0.52) | -0.80  (-1.17;-0.44) |

Model 1 is adjusted for age at baseline and Rotterdam Study Cohort

Model 2 is additionally adjusted for smoking, alcohol use, and TSH

Model 3 is model 2 + additional adjustment for serum cholesterol, serum CRP, body mass index, hypertension, and diabetes

Model 4 is model 2 + additional adjustment for SHBG (only when total testosterone is the exposure)

Model 5 is model 2 + additional adjustment for SMI

Abbreviations: CI, confidence interval; CRP, C-reactive protein; eGFRcreat, estimated glomerular filtration rate (eGFR) based on serum creatinine; eGFRcys, eGFR based on serum cystatin C; n, number; SHBG, sex-hormone binding globulin; SMI, skeletal muscle index; TSH, thyroid-stimulating hormone.

**Supplementary Table S2.** Association of standardized serum free testosterone within the reference range with eGFRcreat and eGFRcys in men and women

| **Subgroup** | **Outcome** | **Beta (95% CI)**  **Model 1** | **Beta (95% CI)**  **Model 2** |
| --- | --- | --- | --- |
| Men | *Reference range defined using 2.5^th^ and 97.5^th^ percentile (0.12-0.47 nmol/L)* | | |
|  | eGFRcreat, baseline (n = 3889) | -0.50 (-1.02;0.01) | -0.48 (-1.00;0.03) |
|  | eGFRcreat, repeated (n = 3889) | -0.29 (-0.77;0.20) | -0.26 (-0.75;0.22) |
|  | eGFRcys, baseline (n = 3889) | 0.90 (0.30;1.50) | 0.92 (0.32;1.51) |
| Women | *Reference range defined using 2.5^th^ and 97.5^th^ percentile (0.003-0.03 nmol/L)* | | |
|  | eGFRcreat, baseline (n = 5119) | -1.07 (-1.76;-0.39) | -1.12 (-1.80;-0.44) |
|  | eGFRcreat, repeated (n = 5119) | -1.17 (-1.83;-0.51) | -1.21 (-1.87;-0.55) |
|  | eGFRcys, baseline (n = 5119) | -1.92 (-2.68;-1.16) | -1.80 (-2.56;-1.05) |

Model 1 is adjusted for age at baseline and Rotterdam Study Cohort

Model 2 is additionally adjusted for smoking, alcohol use, and TSH

Abbreviations: CI, confidence interval; eGFRcreat, estimated glomerular filtration rate (eGFR) based on serum creatinine; eGFRcys, eGFR based on serum cystatin C; n, number; TSH, thyroid-stimulating hormone

**Supplementary Table S3.** Association of standardized serum free testosterone with eGFRcreat and eGFRcys in men and women not using lipid-lowering medication or sex hormones and modulators of the genital system

| **Subgroup** | **Outcome** | **Beta (95% CI)**  **Model 1** | **Beta (95% CI)**  **Model 2** |
| --- | --- | --- | --- |
| Men (n = 3355) | eGFRcreat, baseline | -0.73 (-1.19;-0.27) | -0.74 (-1.20;-0.28) |
|  | eGFRcreat, repeated | -0.50 (-0.93;-0.08) | -0.51 (-0.94;-0.09) |
|  | eGFRcys, baseline | 0.38 (-0.16;0.92) | 0.39 (-0.14;0.93) |
| Women (n = 4365) | eGFRcreat, baseline | -1.11 (-1.50;-0.72) | -1.12 (-1.51;-0.72) |
|  | eGFRcreat, repeated | -0.87 (-1.25;-0.49) | -0.90 (-1.27;-0.52) |
|  | eGFRcys, baseline | -0.86 (-1.29;-0.42) | -0.88 (-1.31;-0.44) |

Model 1 is adjusted for age at baseline and Rotterdam Study Cohort

Model 2 is additionally adjusted for smoking, alcohol use, and TSH

Abbreviations: CI, confidence interval; eGFRcreat, estimated glomerular filtration rate (eGFR) based on serum creatinine; eGFRcys, eGFR based on serum cystatin C; n, number; TSH, thyroid-stimulating hormone

**Supplementary Table S4.** Association of standardized serum SHBG with eGFRcreat and eGFRcys, in men

| **Exposure** | **Outcome** | **Beta (95% CI),**  **Model 1** | **Beta (95% CI),**  **Model 2** |
| --- | --- | --- | --- |
| Serum SHBG  (n = 4095) | eGFRcreat, baseline | 1.57 (1.16;1.98) | 1.49 (1.08;1.90) |
|  | Serum creatinine, baseline | -0.03 (-0.04;-0.02) | -0.03 (-0.04;-0.02) |
|  | eGFRcys, baseline | -0.61 (-1.09;-0.12) | -0.31 (-0.79;0.17) |
|  | Serum cystatin C, baseline | -0.00 (-0.01;0.01) | -0.00 (-0.01;0.00) |

Model 1 is adjusted for age at baseline and Rotterdam Study Cohort

Model 2 is additionally adjusted for smoking, alcohol use, and TSH

Abbreviations: CI, confidence interval; eGFRcreat, estimated glomerular filtration rate (eGFR) based on serum creatinine; eGFRcys, eGFR based on serum cystatin C; n, number; SHBG, sex-hormone binding globulin; TSH, thyroid-stimulating hormone

**Supplementary Table S5.** Association between standardized serum total testosterone with urine ACR at baseline, separately for men and women

| **Subgroup** | **Outcome** | **Beta (95% CI),**  **Model 1** | **Beta (95% CI),**  **Model 2** |
| --- | --- | --- | --- |
| Men (n = 4,095) | Urine ACR | -0.20 (-0.28;-0.12) | -0.21 (-0.29;-0.13) |
| Women (n = 5,389) | Urine ACR | -0.17 (-0.86;0.52) | -0.31 (-0.99;0.38) |

Model 1 is adjusted for age at baseline

Model 2 is additionally adjusted for smoking, alcohol use, and TSH

Abbreviations: ACR, albumin-to-creatinine ratio; CI, confidence interval; n, number; TSH, thyroid-stimulating hormone

**Supplementary Figure S1.** Flowchart of the study selection


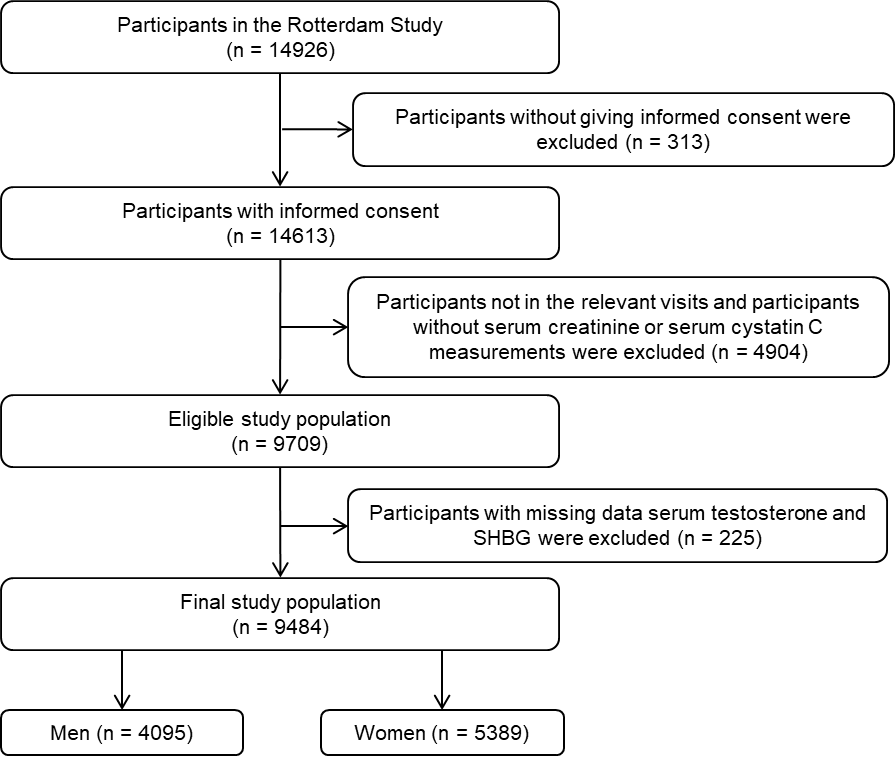


Abbreviations: SHBG, sex-hormone binding globulin.

**STROBE Statement**

|  | | Item No | Recommendation | Page No |
| --- | --- | --- | --- | --- |
| **Title and abstract** | | 1 | (*a*) Indicate the study’s design with a commonly used term in the title or the abstract | 2 |
|  |  |  | (*b*) Provide in the abstract an informative and balanced summary of what was done and what was found | 2 |
| Introduction | | | | |
| Background/rationale | | 2 | Explain the scientific background and rationale for the investigation being reported | 3 |
| Objectives | | 3 | State specific objectives, including any prespecified hypotheses | 3 |
| Methods | | | | |
| Study design | | 4 | Present key elements of study design early in the paper | 4 |
| Setting | | 5 | Describe the setting, locations, and relevant dates, including periods of recruitment, exposure, follow-up, and data collection | 4 |
| Participants | | 6 | (*a*) Give the eligibility criteria, and the sources and methods of selection of participants. Describe methods of follow-up | 4 |
|  |  |  | (*b*) For matched studies, give matching criteria and number of exposed and unexposed | *NA* |
| Variables | | 7 | Clearly define all outcomes, exposures, predictors, potential confounders, and effect modifiers. Give diagnostic criteria, if applicable | 4-7 |
| Data sources/ measurement | | 8* | For each variable of interest, give sources of data and details of methods of assessment (measurement). Describe comparability of assessment methods if there is more than one group | 4-6 |
| Bias | | 9 | Describe any efforts to address potential sources of bias | 6-7 |
| Study size | | 10 | Explain how the study size was arrived at | *NA* |
| Quantitative variables | | 11 | Explain how quantitative variables were handled in the analyses. If applicable, describe which groupings were chosen and why | 6-7 |
| Statistical methods | | 12 | (*a*) Describe all statistical methods, including those used to control for confounding | 6-7 |
|  |  |  | (*b*) Describe any methods used to examine subgroups and interactions | 6-7 |
|  |  |  | (*c*) Explain how missing data were addressed | 7 |
|  |  |  | (*d*) If applicable, explain how loss to follow-up was addressed | *NA* |
|  |  |  | (*e*) Describe any sensitivity analyses | 6-7 |
| Results | | | |  |
| Participants | | 13* | (a) Report numbers of individuals at each stage of study—eg numbers potentially eligible, examined for eligibility, confirmed eligible, included in the study, completing follow-up, and analysed | 8, Supplementary Figure 1 |
|  |  |  | (b) Give reasons for non-participation at each stage | *NA* |
|  |  |  | (c) Consider use of a flow diagram | Supplementary Figure 1 |
| Descriptive data | | 14* | (a) Give characteristics of study participants (eg demographic, clinical, social) and information on exposures and potential confounders | 8, Table 1 |
|  |  |  | (b) Indicate number of participants with missing data for each variable of interest | Table 1 |
|  |  |  | (c) Summarise follow-up time (eg, average and total amount) | 8 |
| Outcome data | | 15* | Report numbers of outcome events or summary measures over time | *NA* |
| Main results | | 16 | (a) Give unadjusted estimates and, if applicable, confounder-adjusted estimates and their precision (eg, 95% confidence interval). Make clear which confounders were adjusted for and why they were included | *8-10* |
|  | |  | (b) Report category boundaries when continuous variables were categorized | *NA* |
|  | |  | (c) If relevant, consider translating estimates of relative risk into absolute risk for a meaningful time period | *NA* |
| Other analyses | | 17 | Report other analyses done—eg analyses of subgroups and interactions, and sensitivity analyses | *8-10* |
| Discussion | | | | |
| Key results | 18 | Summarise key results with reference to study objectives | | 11 |
| Limitations | 19 | Discuss limitations of the study, taking into account sources of potential bias or imprecision. Discuss both direction and magnitude of any potential bias | | 13 |
| Interpretation | 20 | Give a cautious overall interpretation of results considering objectives, limitations, multiplicity of analyses, results from similar studies, and other relevant evidence | | 11-14 |
| Generalisability | 21 | Discuss the generalisability (external validity) of the study results | | 13 |
| Other information | | | | |
| Funding | 22 | Give the source of funding and the role of the funders for the present study and, if applicable, for the original study on which the present article is based | | 15 |

*Give information separately for exposed and unexposed groups.

**Note:** An Explanation and Elaboration article discusses each checklist item and gives methodological background and published examples of transparent reporting. The STROBE checklist is best used in conjunction with this article (freely available on the Web sites of PLoS Medicine at http://www.plosmedicine.org/, Annals of Internal Medicine at http://www.annals.org/, and Epidemiology at http://www.epidem.com/). Information on the STROBE Initiative is available at http://www.strobe-statement.org.
